# Long noncoding RNA CASC7 is a novel regulator of glycolysis in oesophageal cancer via a miR-143-3p-mediated HK2 signalling pathway

**DOI:** 10.1038/s41420-022-01028-y

**Published:** 2022-04-26

**Authors:** Wei Sun, Dao Wang, Yukun Zu, Yu Deng

**Affiliations:** Department of Thoracic Surgery, Tongji Hospital, Huazhong University of Science and Technology, 430030 Wuhan, China

**Keywords:** Cancer metabolism, Long non-coding RNAs

## Abstract

Long noncoding RNAs have been proven to play a crucial role in many tumours. Here, we explored the role of the lncRNA cancer susceptibility candidate 7 (CASC7) in oesophageal cancer. LncRNA CASC7 was identified in our database analysis, and we found that it was significantly higher in oesophageal tumour tissue than in normal tissue and that high expression of lncRNA CASC7 predicted a poor prognosis. Furthermore, we verified through cell experiments that low expression of lncRNA CASC7 in oesophageal cancer cells significantly inhibited tumour proliferation, which could be explained by the effect of lncRNA CASC7 on aerobic glycolysis. Next, we found that the expression of CASC7 and hexokinase 2 (HK2) in oesophageal cancer was positively correlated in database analysis, and this conclusion was further verified in cell experiments. To determine the mechanism, we found that miR-143-3p can bind to both lncRNA CASC7 and HK2. In clinical specimens, we also found high expression of lncRNA CASC7 in tumours, and the expression levels of lncRNA CASC7 and HK2 were positively correlated. In conclusion, downregulating lncRNA CASC7 could inhibit tumour proliferation by reducing glycolysis through the miR-143-3p/HK2 axis.

## Introduction

Oesophageal cancer is one of the most common tumours, and it has a very poor prognosis and extremely high fatality rate; it is the sixth leading cause of cancer-related deaths in the world [1, 2]. To find new therapeutic strategies, it is crucial to determine the molecular mechanisms underlying oesophageal cancer development.

Long noncoding RNAs (lncRNAs) have been a hot research spot in recent years. LncRNAs are a class of RNA molecules more than 200 nt in length [3]. They do not directly participate in encoding proteins, but they do take part in a variety of important regulatory processes, such as chromatin modification, transcriptional activation, transcription interference, and intranuclear transport [3–6]. Here, we identified the lncRNA cancer susceptibility candidate 7 (CASC7) from bioinformatics analysis. Due to its relatively high expression in oesophageal cancer, it is a 9.3 kb lncRNA that has been reported to have tumour-inhibitory functions in glioma, colon cancer, and lung cancer [7–9]. However, no studies have been performed in oesophageal cancer, and how it functions in oesophageal cancer is still a mystery. In this article, we will explore the role it plays in oesophageal cancer.

In recent years, the Warburg effect has become one of the most popular topics in tumour research. Tumour cells can evade the normal apoptotic process through abnormal glucose metabolism behaviour, that is, the Warburg effect, and this is a key factor in the pathogenesis of tumours [10, 11]. The abnormal glucose metabolism behaviour is aerobic glycolysis; even under aerobic conditions, the tumour cells seem to adjust better to glycolysis than oxidative phosphorylation [12]. Hexokinase 2 (HK2) is one of the most important enzymes in the process of glycolysis [13]. HK2 has been proven to be involved in the progression of many tumours, including colon cancer [14], gallbladder cancer [15], glioma [16], myeloma [17], and pancreatic cancer [18], as well as oesophageal cancer, but the detailed mechanism needs to be further studied.

Evidence from other studies has confirmed that lncRNAs are involved in the tumour metabolic regulation process. Whether lncRNA CASC7 is involved in the process of glucose metabolism still needs to be addressed. In this study, we report the correlation between lncRNA CASC7 and HK2 in oesophageal cancer and their roles in this tumour.

## Materials and methods

### Cell lines

We obtained human oesophageal cancer cells (ECA109, TE2, TE3, TE7 and TE8) from the American Type Culture Collection. TE2 and TE7 were cultured in DMEM with high glucose (Invitrogen, CA, USA) containing 10% foetal bovine serum, and these cells were maintained at 37 °C with 5% CO_2_.

### Cell transfection

The shNC, shCASC7#1, and shCASC7#2 lentiviruses were purchased from Genchem, Shanghai. Then, we constructed stable cell lines following the protocol described in another article [19]. HK2 plasmid was purchased from Weizhen, Shandong, and miR-143-3p mimics and miR-143-3p inhibitor were from Genchem, Shanghai. These plasmids were transfected by Lipofectamine 3000 (Invitrogen, CA, USA) following the manufacturer’s instructions.

### CCK8, cell colony formation assay

Three thousand cancer cells from different treatment groups were seeded into each well of a 96-well plate. The cells were cultured for 96 h, and then CCK8 solution was added to each well for 2 h. Eventually, we detected the absorbance of each well at 450 nm.

One hundred cells were seeded into each well from every group on a six-well plate, and the medium was changed regularly. After 2 weeks of incubation, the cells were fixed with 4% paraformaldehyde. Finally, we calculated the colony formation efficiency of each group.

### qRT–PCR analysis

Total RNA was extracted from cancer cells with TRIzol reagent (Invitrogen, CA, USA). Then, RNA was reverse transcribed into complementary DNA (cDNA) using a Superscript Reverse Transcriptase Kit (Transgene, France) following the manufacturer’s protocol. Finally, qRT–PCR was performed with an ABI7300 real-time PCR system (Applied Biosystems) by using a Super SYBR Green Kit (Transgen, France). The relative primers were as follows: CASC7, 5′- TCCACCTAGACCCGACTTTGG-3′ and 5′- GTGTTCCACGATTTCCCTGTT-3′; HK2, 5′-GAGCCACCACTCACCCTACT-3′ and 5′-CCAGGCATTCGGCAATGTG-3′; miR-143-3p, 5′-CTCGCTTCGGCAGCACA-3′ and 5′-AACGCTTCACGAATTTGCGT-3′; and GAPDH, 5′-GAGAGACCCTCACTGCTG-3′ and 5′-GATGGTACATGACAAGGTGC-3′.

### Western blotting assay

Western blotting was performed following standard protocols. The antibodies against GAPDH and HK2 were purchased from Cell Signaling Technology Company (Massachusetts, USA). Goat anti-mouse and anti-rabbit antibodies were used as secondary antibodies (Jackson ImmunoResearch, PA, USA).

### Glucose secretion and lactate production assay

Each well was seeded with 5 * 10^3^ tumour cells from every group on a six-well plate for 24 h, and then the medium was replaced with FBS-free DMEM for another 24 h to starve the cells. Next, we changed the medium to glucose-free medium. After 8 h, the glucose concentration was measured by using the Amplex Red Glucose Assay Kit (Thermo Fisher Scientific, USA) according to the manufacturer’s instructions. The lactate concentration was measured by using a Lactate Assay kit (BioVision, K607-100, Milpitas, CA, USA).

### ECAR analysis

The extracellular acidification rate (ECAR) assay was performed with the Seahorse Extracellular Flux Analyser XF96 (Seahorse Bioscience) according to the manufacturer’s instructions. A total of 8000 cells from each group were seeded into each well on an XF96-well plate for 24 h. Next, the medium was replaced with serum-free DMEM for another 24 h to starve the cells. Then, the medium was replaced with unbuffered DMEM, and glucose, oligomycin and 2-deoxy glucose were added in sequence up to final concentrations of 10 mM, 1 μM, and 50 mM. The ECAR was measured as mpH/min.

### Dual-luciferase reporter gene assay

The cells were seeded in 24-well plates. Then, the cells were cotransfected with the relative plasmid and miRNA. After incubation for 48 h, the cell lysates were collected and tested for luciferase by a dual-luciferase assay system (Promega, USA) following the manufacturer’s instructions.

### RNA fluorescent in situ hybridization (FISH)

Following the instructions of the RNA-FISH kit (BersinBio, Guangzhou, China), probes were added to tumour cells or tissue slices of both normal and tumour tissues. Then, after denaturation and hybridisation, the slices were rinsed and dyed with DAPI, and the results were observed under a fluorescence microscope.

### Statistical analysis

All experimental data were analysed by GraphPad Prism 8.0 software (La Jolla, USA). All experiments in triplicates, Student’s *t* test was performed to analyse the significant difference between every group, and the significance was considered at *P* < 0.05.

## Results

### The lncRNA CASC7 is upregulated and correlated with poor prognosis in oesophageal cancer

**Fig. 1:**
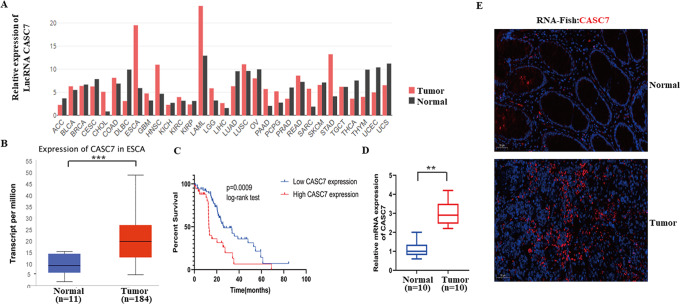
lncRNA CASC7 is upregulated and correlated with poor prognosis in oesophageal cancer. **A** Expression of lncRNA CASC7 in a series of tumours and their adjacent tissues from GEPIA. **B** Expression of lncRNA CASC7 in oesophageal cancer tissues and normal tissues from TCGA analysis. **C** Survival analysis of high-expression and low-expression lncRNA CASC7 in oesophageal cancer from the database. **D** qRT–PCR assay for mRNA expression of lncRNA CASC7 in tumour and normal tissues from surgery patients. (*n* = 10) (**E**). RNA-FISH of lncRNA CASC7 in tumour and normal tissues from surgery patients. Scale bar: 20 μm. **P < 0.01.

To find the potential correlation between lncRNA CASC7 and oesophageal cancer we analysed the GEPIA database, and we found that lncRNA CASC7 was highly expressed in a variety of tumour tissues compared with normal tissues, and the expression of lncRNA CASC7 was particularly high in oesophageal cancer compared to other tumours (Fig. 1A). Similarly, through TCGA database analysis, we found that the expression level of lncRNA CASC7 in oesophageal cancer tissues was significantly higher than that in normal tissues (Fig. 1B). These results indicated that lncRNA CASC7 may be closely related to the progression of oesophageal cancer. Furthermore, through survival analysis, the prognosis of patients with high expression of oesophageal cancer was significantly worse than that of the low expression group (Fig. 1C). To verify this finding, we collected oesophageal cancer tissues and normal tissues from surgery patients, and through qRT–PCR assays and RNA-FISH experiments, we obtained similar results that lncRNA CASC7 was highly expressed in cancer tissues (Fig. 1D, E). Next, we analysed patient characteristics and found that the level of lncRNA CASC7 was closely related to the degree of tumour differentiation and tumour TNM stage (Table 1). The above results indicated that lncRNA CASC7 played an important role in the progression of oesophageal cancer.

**Table 1:** Relationship between the expression level of lncRNA CASC7 and tumour characteristics.

Characteristics	*n*	CASC7 levels		*P* value
		low	high	
Age (years)				0.744
≥50	65	25	40	
<50	22	10	12	
Gender				0.367
male	52	23	29	
female	35	12	23	
Tumour size (cm)				0.380
≥5	46	16	30	
<5	41	19	22	
Differentiation				0.022*
Moderately and highly	38	21	17	
Poorly	49	14	35	
TNM stage				0.016*
I + II	35	20	15	
III + IV	52	15	37	

Low/high by the sample mean. Pearson *χ*^2^ test. *P < 0.05 was considered statistically significant.

### Knockdown of lncRNA CASC7 inhibits the proliferation of oesophageal cancer

**Fig. 2:**
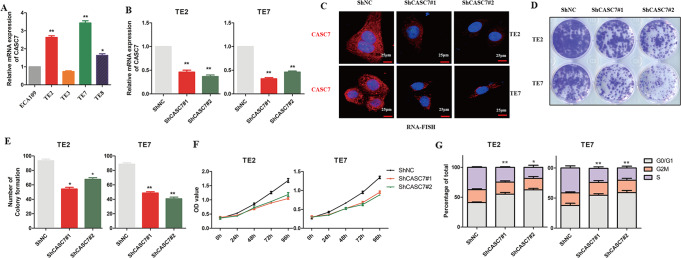
Knockdown of lncRNA CASC7 inhibits proliferation of oesophageal cancer. **A** Relative mRNA expression of lncRNA CASC7 in different oesophageal cancer cell lines (*n* = 3). **B** qRT–PCR assay for lncRNA CASC7 expression in TE2 and TE7 cells after transfection with shCASC7 (*n* = 3). **C** RNA-FISH assay for lncRNA CASC7 expression in TE2 and TE7 cells following treatment with shCASC7. Scale bar: 25 μm. **D**, **E** A cell colony formation assay was performed to analyse the proliferation of TE2 and TE7 cells after knockdown of CASC7. **F** CCK8 was performed to detect the viability of TE2 and TE7 cells after knockdown of CASC7. **G**. A cell cycle assay was performed to determine the effect on the cell cycle following treatment with shCASC7 (*n* = 3). **P < 0.01, *P < 0.05.

To analyse the role of lncRNA CASC7 in oesophageal cancer cells, qRT–PCR was performed to analyse the relative expression level of lncRNA CASC7 in oesophageal cancer cell lines. TE2 and TE7 showed a higher expression level of lncRNA CASC7 (Fig. 2A), which may be related to tumour type or malignant degree. Thus, we silenced lncRNA CASC7 using targeted shRNAs, and qRT–PCR and RNA-FISH assays were conducted to confirm the efficiency of the shRNAs (Fig. 2B, C). Next, we completed a series of experiments to explore the function of lncRNA CASC7 in tumour cells. In the colony formation assay, cells transfected with shRNAs showed fewer colonies (Fig. 2D, E). The CCK8 experiment also revealed similar results showing that downregulating lncRNA CASC7 inhibited the proliferation of oesophageal cancer cells (Fig. 2F). Flow cytometry for the cell cycle assay showed that more tumour cells stayed in G0/G1 phase following treatment with shRNAs (Fig. 2G), which suggested proliferation inhibition. To examine the role of CASC7 in cell death, we performed a cell apoptosis assay to determine the apoptotic cells regulated by CASC7. We found that CASC7 silencing had a slight role in cell apoptosis (Supplemental Fig. 2A). Collectively, these data strongly suggested that knockdown of lncRNA CASC7 inhibits the proliferation of oesophageal cancer.

### Silencing of lncRNA CASC7 attenuates tumour glycolysis in oesophageal cancer

**Fig. 3:**
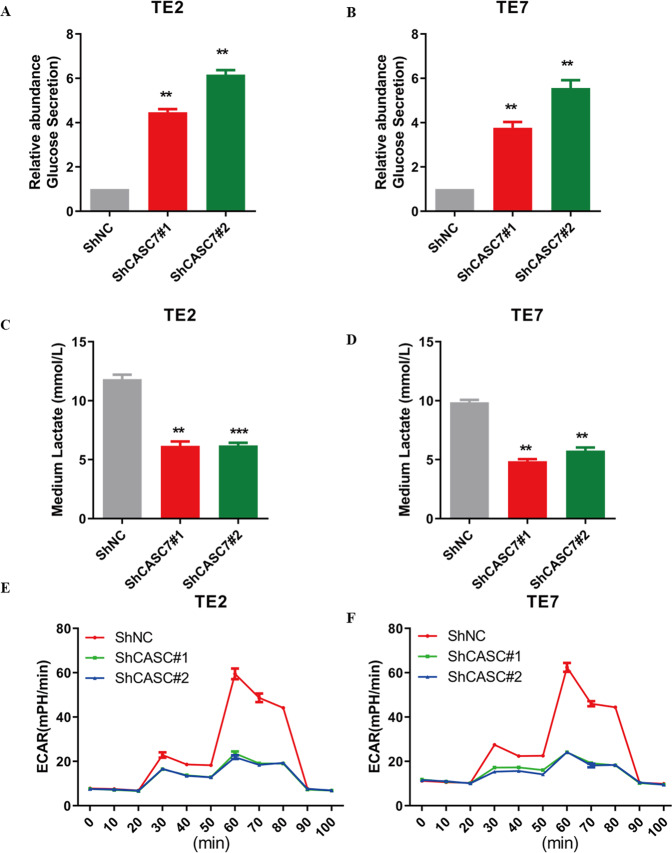
Silencing of lncRNA CASC7 attenuates tumour glycolysis in oesophageal cancer. **A** Relative abundance of glucose secretion levels in the medium of shNC- and shCASC7-transfected TE2 cells (*n* = 3). **B** Relative abundance of glucose secretion levels in the medium of shNC- and shCASC7-transfected TE7 cells (*n* = 3). **C** Lactate concentration level in the medium of shNC- and shCASC7-transfected TE2 cells. **D** Lactate concentration level in the medium of shNC- and shCASC7-transfected TE7 cells (*n* = 3). **E** Extracellular acidification rate (ECAR) analysis presented the glycolytic capacity of shNC- and shCASC7-transfected TE2 and TE7 cells. **F** Extracellular acidification rate (ECAR) analysis presented the glycolytic capacity of shNC- and shCASC7-transfected TE7 cells (*n* = 3). ***P < 0.001, **P < 0.01.

The Warburg effect is one of the characteristics of tumour cells. To determine whether lncRNA CASC7 has an impact on aerobic glycolysis in oesophageal cancer, we conducted a series of experiments. As shown in Fig. 3A, B, cells transfected with shCASC7 secreted glucose levels that were significantly higher than those of the control group, and the production of lactic acid was lower than that of the control group (Fig. 3C, D). That is, knockdown of lncRNA CASC7 could inhibit tumour cell glucose intake and then attenuate glycolysis in tumour cells, eventually producing less lactate. Similarly, the extracellular acidification rate was significantly lower than that in the control group (Fig. 3E, F). Based on these results, lncRNA CASC7 actually played an important role in the process of glycolysis and promoted tumour proliferation by promoting glycolysis of tumour cells.

### LncRNA CASC7 affects tumour glycolysis and proliferation by regulating HK2 expression

**Fig. 4:**
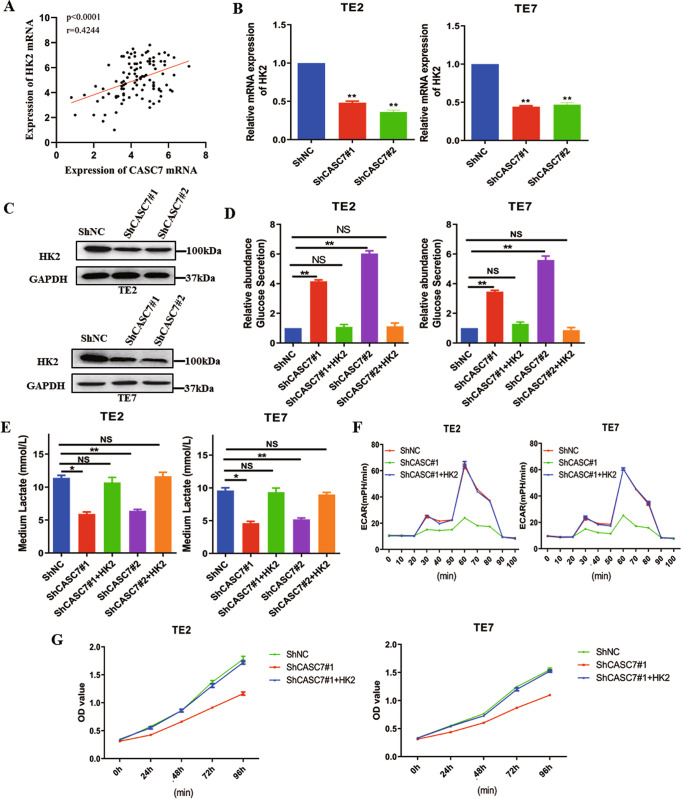
lncRNA CASC7 affects tumour glycolysis and proliferation by regulating HK2 expression. **A** Correlative expression of lncRNA and HK2 in the oesophageal cancer database (cohort 1). **B** qRT–PCR analysis of the relative HK2 mRNA expression in shNC- and shCASC7-transfected TE2 and TE7 cells. **C** Western blotting for HK2 expression in shNC- and shCASC7-transfected TE2 and TE7 cells. **D** Relative abundance of glucose secretion levels in the medium of shNC-transfected, shCASC7-transfected and shCASC7 + HK2-transfected TE2 and TE7 cells (*n* = 3) **E** Lactate concentration level in the medium of shNC-transfected, shCASC7-transfected and shCASC7 + HK2-transfected TE2 and TE7 cells (*n* = 3). **F** Extracellular acidification rate (ECAR) analysis revealed the glycolytic capacity of shNC-transfected, shCASC7-transfected and shCASC7 + HK2-transfected TE2 and TE7 cells. **G** CCK8 assay of shNC-transfected, shCASC7-transfected and shCASC7 + HK2-transfected TE2 and TE7 cells. (*n* = 3) **P < 0.01, *P < 0.05, NS, not significant.

As mentioned previously, HK2 is one of the most important enzymes in the process of glycolysis. To further explore how lncRNA CASC7 affects the process of glycolysis, we obtained from database (cohort 1) analysis that the expression level of lncRNA CASC7 was positively correlated with the expression level of HK2 in oesophageal cancer specimens (Fig. 4A). In oesophageal cancer cell lines, we showed that knockdown of lncRNA CASC7 decreased both the mRNA level and protein level of HK2 (Fig. 4B, C). To further verify our hypothesis, we overexpressed HK2 in shCASC7-transfected cells and found that glucose secretion was significantly reduced (Fig. 4D), lactic acid was obviously increased (Fig. 4E), and the extracellular acidification rate was significantly increased (Fig. 4F), indicating that lncRNA CASC7 affects the process of tumour glycolysis by regulating HK2. Next, we found that in shCASC7 cell lines, high expression of HK2 significantly improved the proliferation of tumour cells (Fig. 4G), which is what we expected. Thus, we concluded that lncRNA CASC7 affects tumour glycolysis and proliferation by regulating HK2 expression.

### LncRNA CASC7 regulates HK2 expression by competitively binding to miR-143-3p

**Fig. 5:**
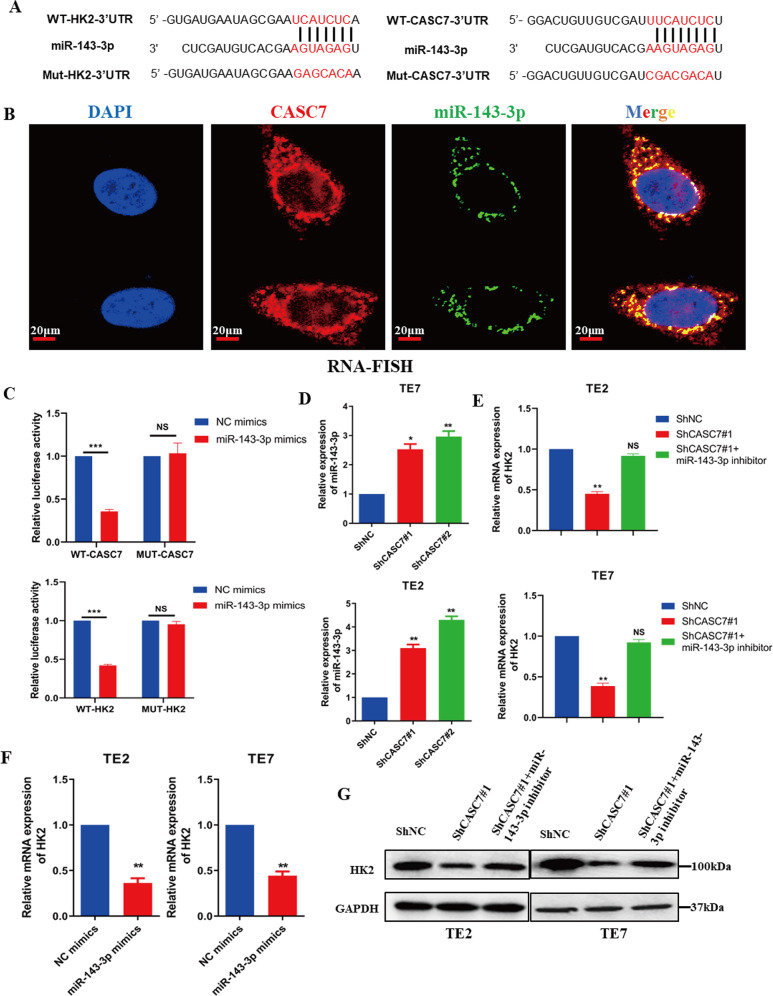
lncRNA CASC7 regulates HK2 expression by competitively binding to miR-143-3p. **A** miR-143-3p has a binding site in CASC7 and HK2 based on analysis of the Diana-lncbase V2 and StarBase databases, and MUT-HK2 and MUT-CASC7 with alterations in the base sequence on the binding sites were designed for further experiments. **B** RNA-FISH assay of lncRNA CASC7 and miR-143-3p colocalization in TE2 cells. Scale bar: 20 μm. **C** A dual-luciferase reporter gene assay was conducted to verify the targeted relationship between miR-143-3p and lncRNA CASC7, as well as miR-143-3p and HK2. **D** qRT–PCR analysis of the relative miR-143-3p mRNA expression in shNC- and shCASC7-transfected TE2 and TE7 cells (*n* = 3). **E** qRT–PCR analysis of the relative HK2 mRNA expression in shNC-transfected, shCASC7-transfected and shCASC7+miR-143-3p inhibitor-transfected TE2 and TE7 cells (*n* = 3). **F** qRT–PCR analysis of relative HK2 mRNA expression in NC mimic- and miR-143-3p mimic-transfected TE2 and TE7 cells (*n* = 3). **G** Western blotting for HK2 expression in shNC-transfected, shCASC7-transfected and shCASC7+miR-143-3p inhibitor-transfected TE2 and TE7 cells (*n* = 3). ***P < 0.001, **P < 0.01, *P < 0.05, NS, not significant.

The mechanism by which lncRNAs act in tumours is very complex and has not yet been fully understood, one mechanism involves lncRNAs affecting the expression of their target genes by regulating miRNAs [20, 21]. We found that miR-143-3p had binding sites in lncRNA CASC7 and HK2 from the Diana-lncbase V2 and StarBase databases (Fig. 5A). To confirm this result, an RNA-FISH experiment was carried out to verify the colocalization of lncRNA CASC7 and miR-143-3p in TE2 cells (Fig. 5B). Similarly, a luciferase assay also found that miR-143-3p interacted with both lncRNA CASC7 and HK2, but it did not bind to MUT-HK2 and MUT-CASC7 (Fig. 5C). Based on this result, we suspected that miR-143-3p might be the bridge between lncRNA CASC7 and HK2. To confirm our hypothesis, through qRT–PCR assays we found that the expression of miR-143-3p significantly increased upon the low expression of CASC7 (Fig. 5D), and correspondingly, the expression of HK2 significantly decreased upon the high expression of miR-143-3p in tumour cells (Fig. 5F). However, the expression level of HK2 was not significantly decreased when miR-143-3p was downregulated at the same time as transfection with shCASC7 (Fig. 5E, G). In addition, we further explored the role of miR-143-3p in cell proliferation and glucose metabolism. Consistent with a previous study, high expression of miR-143-3p resulted in cell proliferation inhibition (Supplementary Fig. 2A), and it negatively regulated glycolysis in tumour cells (Supplementary Fig. 2B–D). In conclusion, downregulation of lncRNA CASC7 inhibited tumour proliferation by reducing glycolysis in tumour cells through the miR-143-3p/HK2 pathway.

### Clinical significance of lncRNA CASC7 and HK2 in oesophageal cancer

**Fig. 6:**
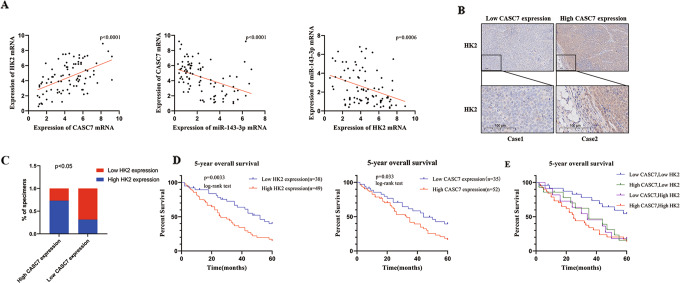
Clinical significance of lncRNA CASC7 and HK2 in oesophageal cancer. **A** Correlative mRNA expression of lncRNA CASC7, HK2 and miR-143-3p in the oesophageal cancer database (cohort 2). **B** Immunohistochemical analysis of the expression of HK2 in low CASC7 expression tumour tissue and high CASC7 expression tumour tissue. Scale bar: 100 μm. **C** Correlative mRNA expression between lncRNA CASC7 and HK2 in tumour tissues (cohort 2) (*n* = 87). **D**, **E** Five-year overall survival assay for different levels of CASC7 and HK2 expression in the oesophageal cancer database (cohort 2) (*n* = 87) ***P < 0.001, *P < 0.05.

In an effort to investigate the role of lncRNA CASC7 and HK2 in our clinical centre, after the consent of the patients, we collected the surgical specimens of patients with oesophageal cancer (cohort 2). We detected the mRNA levels of lncRNA CASC7, HK2 and miR-143-3p in tumour tissues. We found that the tissues with high expression of lncRNA CASC7 were more inclined to have high expression of HK2, and miR-143-3p was negatively correlated with both lncRNA CASC7 and HK2 (Fig. 6A). Further analysis revealed that high lncRNA CASC7 expression in tumours seemed to correspond to high expression of HK2 (Fig. 6C), and this phenomenon was verified by immunohistochemical analysis (Fig. 6B). These results were consistent with the findings previously mentioned. Additionally, a five-year follow-up was conducted. According to the follow-up data, we analysed the 5-year overall survival of these patients. There was no coincidence that patients with high expression of lncRNA CASC7 and HK2 had a poor prognosis (Fig. 6D), and patients with both low expression of lncRNA CASC7 and HK2 had a relatively good prognosis (Fig. 6E). That is, in our clinical data, lncRNA CASC7 and HK2 were also poor predictive prognostic markers.

## Discussion

Despite the continuous improvement of medical research, the treatment of tumours is still an intractable problem. Oesophageal cancer is only one tumour among many, but the mortality rate related to oesophageal cancer ranks at the forefront, with an overall 5-year survival rate of only ~20% [22, 23]. It is extremely urgent to explore the pathogenesis of oesophageal cancer and to find new treatment strategies. In recent years, research on lncRNAs in tumours has become popular. They were initially considered to be the ‘noise’ of genome transcription without biological function; however, lncRNAs have now been proven to play an important role in the development of tumours as important regulatory factors of target genes [24] because they are involved in a series of signalling pathways [25, 26] as either oncogenes or tumour suppressor genes [27]. We identified the lncRNA CASC7 from the database, and its expression in specimens of oesophageal tumour volume was much higher than that in normal tissue. Although lncRNA CASC7 in many tumours plays the role of a tumour suppressor gene, it is a catalyst in oesophageal tumour progression because high lncRNA CASC7 expression indicates a shorter survival time from database analysis. We also found that the expression of lncRNA CASC7 was higher in tumour tissues than in normal tissues collected from surgery patients, and it was strongly related to the degree of tumour differentiation and tumour TNM stage. Next, we verified this conclusion through cell experiments. We found that the proliferation of tumour cells was significantly inhibited after the downregulation of lncRNA CASC7, indicating that lncRNA CASC7 played an important role in the progression of tumours.

In the 1920s, Warburg found that glucose metabolism in tumour cells was tenfold higher than that in nontumor cells, and the level of lactic acid also increased significantly. Subsequently, he found that the survival of tumour cells depended on the supply of glucose and oxygen [28]. In short, aerobic glycolysis is the characteristic metabolic mode of tumours. The effect of aerobic glycolysis on tumours is multifaceted. It is not only a rapid way to produce ATP [29] but can also promote tumour biosynthesis [30, 31], even changing the tumour microenvironment to be more suitable for tumour progression [32, 33] and influencing cell signal transduction [34, 35]. In recent years, a series of studies have shown that lncRNAs can influence aerobic glycolysis [36–38], providing an idea for our study. Results from our experiments indicated that downregulation of lncRNA CASC7 increased the formation of glucose and increased lactic acid levels, and even the extracellular acidification rate was clearly inhibited, illustrating that low expression of lncRNA CASC7 can inhibit tumour aerobic glycolysis.

How does lncRNA CASC7 affect glycolysis in oesophageal cancer cells? We speculated that lncRNA CASC7 can regulate the expression of key enzymes in the glycolysis process. HK2, PKM2, GLUT1 and PFK1 are important enzymes in the process of glycolysis [39, 40]. From database analysis, we found that in oesophageal tumours, lncRNA CASC7 positively correlated with the expression of HK2, and it was verified by cell experiments that downregulation of lncRNA CASC7 could reduce the expression of HK2. Then, we transfected the HK2 plasmid into low-expressing lncRNA CASC7 tumour cells and found that the inhibition of aerobic glycolysis decreased significantly. These findings suggested that lncRNA CASC7 affects tumour glycolysis by regulating HK2. LncRNAs can adsorb miRNAs through base pairing, resulting in loss or decrease of miRNA function. It has been reported that lncRNA CASC7 can interact with several microRNAs, such as miR-21 [41], miR-10a [42], miR-30c [43] and miR-92a [9]. To find the microRNA that can act as the bridge between lncRNA CASC7 and HK2, we searched the Diana-lncbase V2 and StarBase databases and found that miR-143-3p was worthy of further investigation. Next, we carried out a series of experiments, which confirmed that miR-143-3p was the downstream target of lncRNA CASC7 and could regulate HK2 expression.

To further confirm our conclusion, we collected specimens from clinical surgery patients. Then, we conducted qRT–PCR and immunohistochemistry experiments, and there was no doubt that the expression of HK2 was positively related to lncRNA CASC7 expression. Furthermore, we found that low expression of lncRNA CASC7 and HK2 predicted a relatively good prognosis. These results were consistent with our previous conclusion.

The pathogenic mechanism and development of tumours are multifactorial; that is, tumour therapy will always be a difficult challenge. In the future, lncRNA CASC7 may provide a new clue to explore the treatments of oesophageal cancer.

## Supplementary information

lncRNA CASC7 have slight role in cell apoptotic of oesophageal cancer

High expression of miR-143-3p inhibits the proliferation of oesophageal cancer and attenuates tumour glycolysis in oesophageal cancer

Supplemental figure legends

AJE editing certificate

Original Data File

## Data Availability

The datasets used and/or analysed during the current study are available from the corresponding author on reasonable request.

## References

[1] Arnal MJD (2015). Esophageal cancer: risk factors, screening and endoscopic treatment in Western and Eastern countries. World J Gastroenterol.

[2] Bollschweiler E, Plum P, Mönig SP, Hölscher AH (2017). Current and future treatment options for esophageal cancer in the elderly. Expert Opin Pharmacol..

[3] Huang Y (2018). The novel regulatory role of lncRNA-miRNA-mRNA axis in cardiovascular diseases. J Cell Mol Med.

[4] Xu J, Bai J, Zhang X, Lv Y, Gong Y, Liu L (2017). A comprehensive overview of lncRNA annotation resources. Brief Bioinform.

[5] Sun W, Shen NM, Fu SL (2019). Involvement of lncRNA-mediated signaling pathway in the development of cervical cancer. Eur Rev Med Pharm Sci.

[6] Fang Y, Fullwood MJ (2016). Roles, functions, and mechanisms of long non-coding RNAs in cancer. Genom Proteom Bioinforma.

[7] Gong X, Liao X, Huang M (2019). LncRNA CASC7 inhibits the progression of glioma via regulating Wnt/beta-catenin signaling pathway. Pathol Res Pract.

[8] Zhang Z, Fu C, Xu Q, Wei X (2017). Long non-coding RNA CASC7 inhibits the proliferation and migration of colon cancer cells via inhibiting microRNA-21. Biomed Pharmacother.

[9] Chen L, Li X, Lu C, Zhao Y, Zhu J, Yang L (2020). The long noncoding RNA CASC7 inhibits growth and invasion of nonsmall cell lung cancer cells through phosphatase and tensin homolog upregulation via sequestration of miR92a. Int J Oncol.

[10] Liberti MV, Locasale JW (2016). The Warburg effect: how does it benefit cancer cells?. Trends Biochem Sci.

[11] Spencer NY, Stanton RC (2019). The Warburg effect, lactate, and nearly a century of trying to cure cancer. Semin Nephrol.

[12] Lunt SY, Vander HM (2011). Aerobic glycolysis: meeting the metabolic requirements of cell proliferation. Annu Rev Cell Dev Biol.

[13] Garcia SN, Guedes RC, Marques MM (2019). Unlocking the potential of HK2 in cancer metabolism and therapeutics. Curr Med Chem.

[14] Li H, Lu S, Chen Y, Zheng L, Chen L, Ding H (2019). AKT2 phosphorylation of hexokinase 2 at T473 promotes tumorigenesis and metastasis in colon cancer cells via NF-kappaB, HIF1alpha, MMP2, and MMP9 upregulation. Cell Signal.

[15] Chen J, Yu Y, Li H, Hu Q, Chen X, He Y (2019). Long non-coding RNA PVT1 promotes tumor progression by regulating the miR-143/HK2 axis in gallbladder cancer. Mol Cancer.

[16] Zhang B, Chen J, Cui M, Jiang Y (2020). LncRNA ZFAS1/miR-1271-5p/HK2 promotes glioma development through regulating proliferation, migration, invasion and apoptosis. Neurochem Res.

[17] Gu Z, Xia J, Xu H, Frech I, Tricot G, Zhan F (2017). NEK2 promotes aerobic glycolysis in multiple myeloma through regulating splicing of pyruvate kinase. J Hematol Oncol.

[18] Jiang SH, Dong FY, Da LT, Yang XM, Wang XX, Weng JY (2020). Ikarugamycin inhibits pancreatic cancer cell glycolysis by targeting hexokinase 2. FASEB J.

[19] Chen C, Wei M, Wang C, Sun D, Liu P, Zhong X (2020). Long noncoding RNA KCNQ1OT1 promotes colorectal carcinogenesis by enhancing aerobic glycolysis via hexokinase-2. Aging (Albany NY).

[20] Yamamura S, Imai-Sumida M, Tanaka Y, Dahiya R (2018). Interaction and cross-talk between non-coding RNAs. Cell Mol Life Sci.

[21] Cao C, Sun J, Zhang D, Guo X, Xie L, Li X (2015). The long intergenic noncoding RNA UFC1, a target of MicroRNA 34a, interacts with the mRNA stabilizing protein HuR to increase levels of beta-catenin in HCC cells. Gastroenterology..

[22] Malhotra A, Sharma U, Puhan S, Chandra BN, Kharb A, Arifa PP (2019). Stabilization of miRNAs in esophageal cancer contributes to radioresistance and limits efficacy of therapy. Biochimie..

[23] Kelly RJ (2019). Emerging multimodality approaches to treat localized esophageal cancer. J Natl Compr Canc Netw.

[24] Bhan A, Soleimani M, Mandal SS (2017). Long noncoding RNA and cancer: a new paradigm. CANCER Res..

[25] Peng WX, Koirala P, Mo YY (2017). LncRNA-mediated regulation of cell signaling in cancer. Oncogene..

[26] Ma Y, Zhang J, Wen L, Lin A (2018). Membrane-lipid associated lncRNA: a new regulator in cancer signaling. Cancer Lett.

[27] Baldassarre A, Masotti A (2012). Long non-coding RNAs and p53 regulation. Int J Mol Sci.

[28] Warburg O, Wind F, Negelein E (1927). The metabolism of tumors in the body. J Gen Physiol.

[29] Shestov AA, Liu X, Ser Z, Cluntun AA, Hung YP, Huang L (2014). Quantitative determinants of aerobic glycolysis identify flux through the enzyme GAPDH as a limiting step. Elife.

[30] Dang CV (2012). Links between metabolism and cancer. Genes Dev.

[31] Boroughs LK, DeBerardinis RJ (2015). Metabolic pathways promoting cancer cell survival and growth. Nat Cell Biol.

[32] Estrella V, Chen T, Lloyd M, Wojtkowiak J, Cornnell HH, Ibrahim-Hashim A (2013). Acidity generated by the tumor microenvironment drives local invasion. Cancer Res.

[33] Gatenby RA, Gawlinski ET (1996). A reaction-diffusion model of cancer invasion. Cancer Res.

[34] Wellen KE, Thompson CB (2010). Cellular metabolic stress: considering how cells respond to nutrient excess. Mol Cell.

[35] Hamanaka RB, Chandel NS (2011). Cell biology. Warbg effest and redox balance. Science.

[36] Xia M, Feng S, Chen Z, Wen G, Zu X, Zhong J (2020). Non-coding RNAs: key regulators of aerobic glycolysis in breast cancer. Life Sci.

[37] Kong XZ, Hu SS, Sun Z, Zuo LH, Kang J, Zhu ZF (2016). Regulation of aerobic glycolysis by long non-coding RNAs in cancer. Biochem Biophys Res Commun.

[38] Liu H, Luo J, Luan S, He C, Li Z (2019). Long non-coding RNAs involved in cancer metabolic reprogramming. Cell Mol Life Sci.

[39] Knobloch TJ, Ryan NM, Bruschweiler-Li L, Wang C, Bernier MC, Somogyi A (2019). Metabolic regulation of glycolysis and AMP activated protein kinase pathways during black raspberry-mediated oral cancer chemoprevention. Metabolites.

[40] Bonatelli M, Silva E, Carcano FM, Zaia MG, Lopes LF, Scapulatempo-Neto C (2019). The Warburg effect is associated with tumor aggressiveness in testicular germ cell tumors. Front Endocrinol.

[41] Liu JH, Li C, Zhang CH, Zhang ZH (2020). LncRNA-CASC7 enhances corticosteroid sensitivity via inhibiting the PI3K/AKT signaling pathway by targeting miR-21 in severe asthma. Pulmonology.

[42] Zhou X, Lu H, Li F, Han L, Zhang H, Jiang Z (2020). LncRNA cancer susceptibility candidate (CASC7) upregulates phosphatase and tensin homolog by downregulating miR-10a to inhibit neuroblastoma cell proliferation. Neuroreport..

[43] Xu YL, Liu Y, Cai RP, He SR, Dai RX, Yang XH (2020). Long non-coding RNA CASC7 is associated with the pathogenesis of heart failure via modulating the expression of miR-30c. J Cell Mol Med.

