# Supplementary figures and images for: Long noncoding RNA CASC7 is a novel regulator of glycolysis in oesophageal cancer via a miR-143-3p-mediated HK2 signalling pathway
Source: Cell Death Discov. 2022 Apr 26;8:231. doi: 10.1038/s41420-022-01028-y (PMC9043207; doi:10.1038/s41420-022-01028-y)

Supplemental Figure2

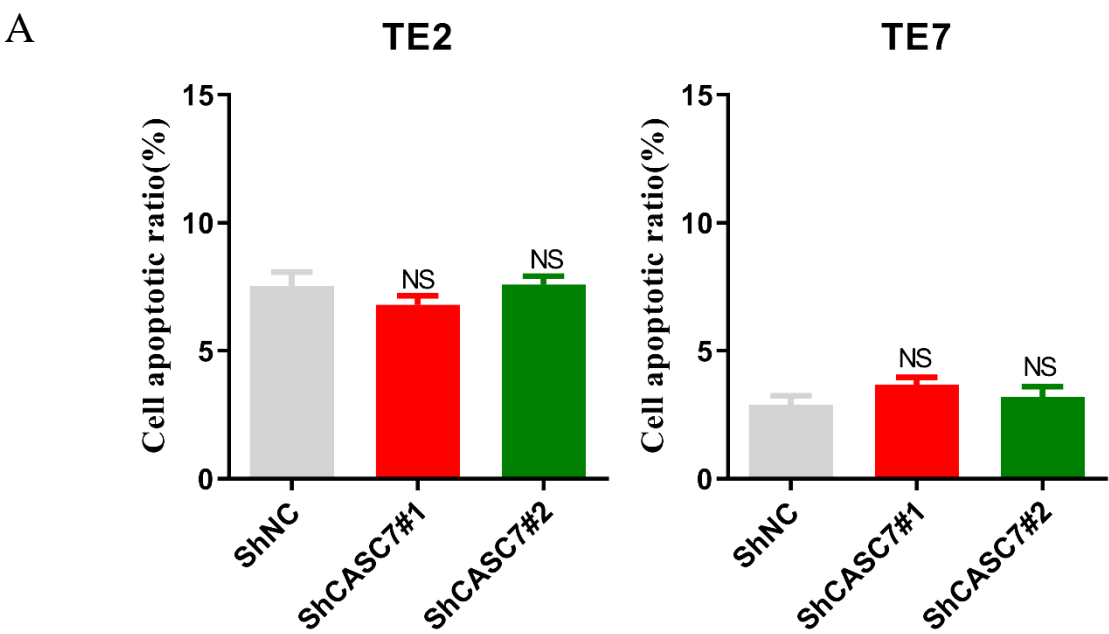

Supplement: Supplementary file 1 — lncRNA CASC7 have slight role in cell apoptotic of oesophageal cancer [file 41420_2022_1028_MOESM1_ESM.pdf]

Supplemental Figure5

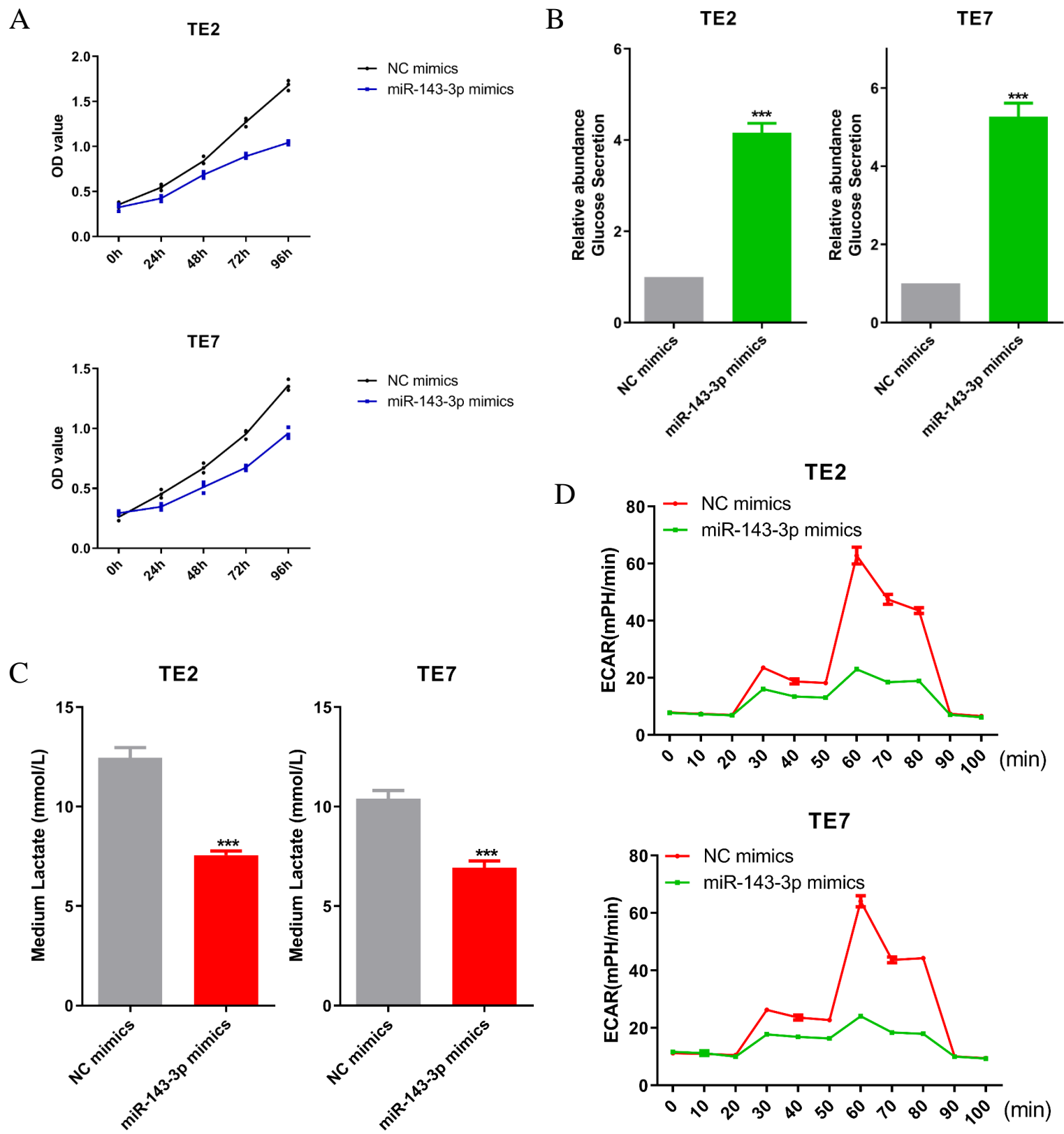

Supplement: Supplementary file 2 — High expression of miR-143-3p inhibits the proliferation of oesophageal cancer and attenuates tumour glycolysis in oesophageal cancer [file 41420_2022_1028_MOESM2_ESM.pdf]
