# Supplementary material for: Long noncoding RNA CASC7 is a novel regulator of glycolysis in oesophageal cancer via a miR-143-3p-mediated HK2 signalling pathway
Source: Cell Death Discov. 2022 Apr 26;8:231. doi: 10.1038/s41420-022-01028-y (PMC9043207; doi:10.1038/s41420-022-01028-y)
Supplement: Supplementary file 3 — Supplemental figure legends [file 41420_2022_1028_MOESM3_ESM.pdf]

Supplemental Figure 2. lncRNA CASC7 have slight role in cell apoptotic of oesophageal cancer

A. Cell apoptosis assay was performed to study the effect of shCASC7 on TE2 and TE7 cells. (n=3). NS, not significant.

Supplemental Figure 5. High expression of miR-143-3p inhibits the proliferation of oesophageal cancer and attenuates tumour glycolysis in oesophageal cancer

A. CCK8 assay was performed to detect the cell viability of TE2 and TE7 cells after transfected with miR-143-3p mimics. (n=3) B. Relative abundance of Glucose secretion level in the medium of miR-134-3p mimics-transfected TE2 and TE7 cells. (n=3). C. Lactate concentration level in the medium of TE2 and TE7 cells following the treatment with miR-143-3p. (n=3) D. Extracellular acidification rate (ECAR) analysis presented the glycolytic capacity of TE2 and TE7 cells following the treatment with miR-143-3p mimics. (n=3). ***$P < 0.001$
