# Supplementary material for: Long noncoding RNA CASC7 is a novel regulator of glycolysis in oesophageal cancer via a miR-143-3p-mediated HK2 signalling pathway
Source: Cell Death Discov. 2022 Apr 26;8:231. doi: 10.1038/s41420-022-01028-y (PMC9043207; doi:10.1038/s41420-022-01028-y)
Supplement: Supplementary file 4 — AJE editing certificate [file 41420_2022_1028_MOESM4_ESM.pdf]

Editing Certificate

This document certifies that the manuscript

Long noncoding RNA CASC7 is a novel regulator of glycolysis in oesophageal cancer via a miR-143-3p-mediated HK2 signalling pathway

prepared by the authors

Wei Sun, Dao Wang, Yukun Zu, Yu Deng

was edited for proper English language, grammar, punctuation, spelling, and overall style by one or more of the highly qualified native English speaking editors at AJE.

This certificate was issued on **March 31, 2022** and may be verified on the AJE website using the verification code **BFBE-D8B4-3F74-E17A-2D7D**.

Neither the research content nor the authors' intentions were altered in any way during the editing process. Documents receiving this certification should be English-ready for publication; however, the author has the ability to accept or reject our suggestions and changes. To verify the final AJE edited version, please visit our verification page at aje.com/certificate. If you have any questions or concerns about this edited document, please contact AJE at support@aje.com.

AJE provides a range of editing, translation, and manuscript services for researchers and publishers around the world. For more information about our company, services, and partner discounts, please visit aje.com.
