# Supplementary material for: Long noncoding RNA CASC7 is a novel regulator of glycolysis in oesophageal cancer via a miR-143-3p-mediated HK2 signalling pathway
Source: Cell Death Discov. 2022 Apr 26;8:231. doi: 10.1038/s41420-022-01028-y (PMC9043207; doi:10.1038/s41420-022-01028-y)
Supplement: Supplementary file 5 — Original Data File [file 41420_2022_1028_MOESM5_ESM.pptx]

## Slide 1
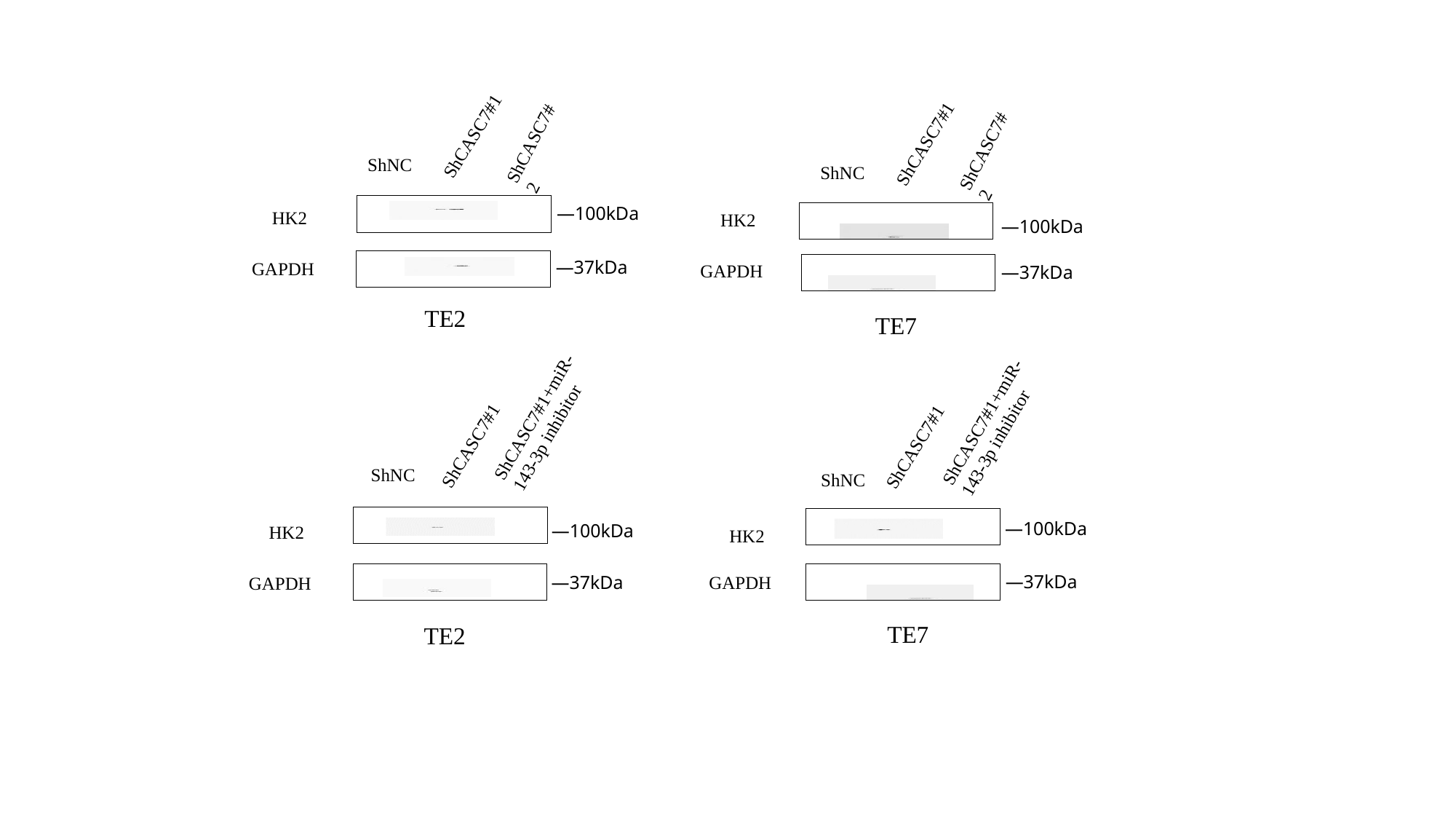

ShCASC7#1
ShCASC7#2
ShNC
HK2
GAPDH
TE2
—100kDa
—37kDa
ShCASC7#1
ShCASC7#2
ShNC
HK2
GAPDH
TE7
—100kDa
—37kDa
ShCASC7#1+miR-143-3p inhibitor
ShCASC7#1
ShNC
HK2
GAPDH
TE2
—100kDa
—37kDa
ShCASC7#1+miR-143-3p inhibitor
ShCASC7#1
ShNC
HK2
GAPDH
TE7
—100kDa
—37kDa
